# Iodine Biofortification of Four *Brassica* Genotypes is Effective Already at Low Rates of Potassium Iodate

**DOI:** 10.3390/nu11020451

**Published:** 2019-02-21

**Authors:** Maria Gonnella, Massimiliano Renna, Massimiliano D’Imperio, Pietro Santamaria, Francesco Serio

**Affiliations:** 1 Institute of Sciences of Food Production, CNR—National Research Council of Italy, Via Amendola 122/D, 70126 Bari, Italy; maria.gonnella@ispa.cnr.it (M.G.); massimiliano.dimperio@ispa.cnr.it (M.D.); francesco.serio@ispa.cnr.it (F.S.); 2 Department of Agricultural and Environmental Science, University of Bari Aldo Moro, Via Amendola 165/A, 70126 Bari, Italy; pietro.santamaria@uniba.it

**Keywords:** curly kale, broccoli raab, mizuna, red mustard, cooked vegetables, steaming, boiling

## Abstract

The use of iodine-biofortified vegetables may be a health alternative instead of iodine-biofortified salt for preventing iodine (I) deficiency and related human disorders. In this study, four *Brassica* genotypes (broccoli raab, curly kale, mizuna, red mustard) were hydroponically grown with three I-IO_3_^−^ rates (0, 0.75 and 1.5 mg/L) to produce iodine-biofortified vegetables. Crop performances and quality traits were analyzed; iodine content was measured on raw, boiled, and steamed vegetables. The highest I rate generally increased I content in all *Brassica* genotypes, without plants toxicity effects in terms of reduced growth or morphological symptoms. After 21 day-iodine biofortification, the highest I content (49.5 µg/100 g Fresh Weight (FW)) was reached in broccoli raab shoots, while after 43 day-iodine biofortification, genotype differences were flattened and the highest I content (66 µg/100 g FW, on average) was obtained using 1.5 mg I-IO_3_/L. Nitrate content (ranging from 1800 to 4575 mg/kg FW) was generally higher with 0.75 mg I-IO_3_/L, although it depended on genotypes. Generally, boiling reduced iodine content, while steaming increased or left it unchanged, depending on genotypes. Applying low levels of I proved to be suitable, since it could contribute to the partial intake of the recommended dose of 150 µg/day: A serving size of 100 g may supply on average 24% of the recommended dose. Cooking method should be chosen in order to preserve and/or enhance the final I amount.

## 1. Introduction

Iodine (I) is an essential trace element in human nutrition, since, as component of thyroid hormones (triiodothyronine and thyroxine), its deficiency is the cause of altered functionality of the thyroid gland [1]. The Recommended Daily Allowance (RDA) value for I is 150 µg/day for adults [2]. An international program of monitoring and prevention of I deficiency commenced in 1993 and aims to reach adequate level of I intake in the global population. The main action of prevention is I fortification of alimentary salt. In two decades, from 1993 to 2013, the policy of salt iodization brought about a reduction in the number of I deficient countries from 110 to 31 [2]. However, at the same time, all countries try to apply the recommendation by World Health Organization to reduce salt dietary intake to 5 g/day in adults. For this reason and in the cases where salt iodization is not efficient, for example in Asian countries where salt is not the main dietary condiment, alternative strategies to increase I intake in the human diet are to be implemented. Food fortification can be a good alternative [3]. Natural I sources in human diet are fish and crustaceans, then milk, eggs, meat, and, at last, vegetables and fruits at lower concentration, depending on the I amount in the soil [4]. In circumstances limiting or avoiding animal derived food or for groups of consumers who exclude some food categories (vegetarians and vegans, people with intolerance or allergies, people who dislikes fish/other food, inhabitants of inland regions), biofortification of some vegetables could be a good and healthy alternative source of food I and, at the same time, a challenge to face the inadequacy of vegetable intake in the Western diet [5]. *Brassica* vegetables are suitable to reach this goal due to the consumption of a large part of the plant (stem, leaves, and inflorescences) and their richness in natural beneficial compounds, mainly antioxidants, which together with an increased I content can confer an added value to their consumption. Moreover, *Brassica* are largely consumed in Asian countries, as well as in Italy, where an adequate level of I in the diet has not yet reached [6]. Hence, I biofortification in *Brassica* species can balance the potential inefficacy of salt iodization. Curly kale and broccoli raab are local widespread genotypes, while mizuna and red mustard are Asian genotypes recently marketed globally for baby leaf or leaf products. The first two species can be eaten only cooked (in broccoli raab, the edible portion is made of leaves, stem, and inflorescence), while the other two can be also used as raw baby leaf in salads.

Leaves are the second organ in the plant, after root, in order of accumulation of I [7]. Iodine biofortification of leafy vegetables was obtained in spinach [8,9], lettuce [10,11,12], and celery [8,13]. Among *Brassicaceae*, some studies of cabbage and Chinese cabbage [7], pak choi [14], and radish [15] have been carried out. In an I biofortification program, food safety is a non-negotiable point in order to avoid the risk of exceeding the I tolerable upper intake level (500–600 µg/day) in the diet [4] and, at the same time, to avoid limits to the consumption of biofortified vegetables. In this regard, two opposite examples can be reported based on the I rate and the applied biofortification protocol: On the one hand, only 25% of the RDA was obtained with 50 g serving of lettuce grown without soil at very low I rates (129 µg/L) [11]; on the other hand, more than 200% of the RDA was obtained with the same serving of spinach grown in soil at a higher I supply (0.4 g/L) [16].

On the other hand, to our knowledge, no one has studied the effect of cooking treatment on the I content in *Brassicaceae* species. Cooking process is known to reduce I accumulated in leaves after biofortification [17,18,19]. In some studies, boiling had different effects on I content, based on the specific characteristics of the process and the vegetable matrix [17,20]. It is, therefore, advisable to assess the final content of I in a biofortified vegetable after the cooking process.

In this study, the main goal was the biofortification of *Brassica* leafy vegetables with low rates of I. Furthermore, since the consumption of some *Brassica* is predominantly cooked, the secondary goal was to assess the intake of I from biofortified *Brassica* after boiling and steaming were applied as cooking methods.

## 2. Materials and Methods 

### 2.1. Plant Material and Cultivation

The experimental trial was carried out in a plastic (polymethacrylate) greenhouse located in Mola di Bari (at “La Noria” experimental farm of the Institute of Sciences of Food Production of the Italian National Research Council; 24 m above sea level; 41°03′ N, 17°04′ E). Four genotypes belonging to the genus *Brassica* were compared: *Brassica rapa* L. (broccoli raab) (seeds from local growers), *Brassica oleracea* L. var. *acephala* (curly kale) (seeds from local growers), *Brassica juncea* var. *japonica* (mizuna) (Riccardo Larosa Sementi, Andria, BAT, Italy), and *Brassica juncea* (red mustard) (Riccardo Larosa Sementi, Andria, BAT, Italy).

*Brassica* seedlings were transplanted on 6 November 2017 at three-four true leaf stage, into pots containing 4.5 L of a perlite:peat mixture in a 3:1 volume ratio and received a nutrient solution (NS) containing (all element concentrations in mM) nitrogen (8 as N-NO_3_^−^ plus 2 as N-NH_4_^+^), phosphorus (1.6), potassium (5.1), magnesium (1.2), calcium (2.5), iron (0.023), copper (0.001), manganese (0.004), zinc (0.002), boron (0.025), and molybdenum (0.0005). The experimental treatments were arranged according to a split-plot design with three replications, where three NSs, with different iodine levels (0, 0.75 and 1.5 mg/L equivalent to 0, 5.9 and 11.8 µM from KIO_3_), were set in the main plots and genotypes in the sub-plots. Pots were arranged on trough-benches (with a slope of 2%) containing 20 plants (one/pot), with each elementary unit made by ten pots. The distance between the pots was 20 cm within the row and 100 cm between rows, with a final density of five plants/m^2^. Each pot was supplied with the NS through drip irrigation emitters (one/pot) set to one two-minute distribution per day. The NS was not recirculated but managed in an open cycle system. Plants were supplied with not-differentiated full strength NSs till 18 December 2017, when the two levels of I were supplied to the plants, except for the untreated controls.

**Figure 1:**
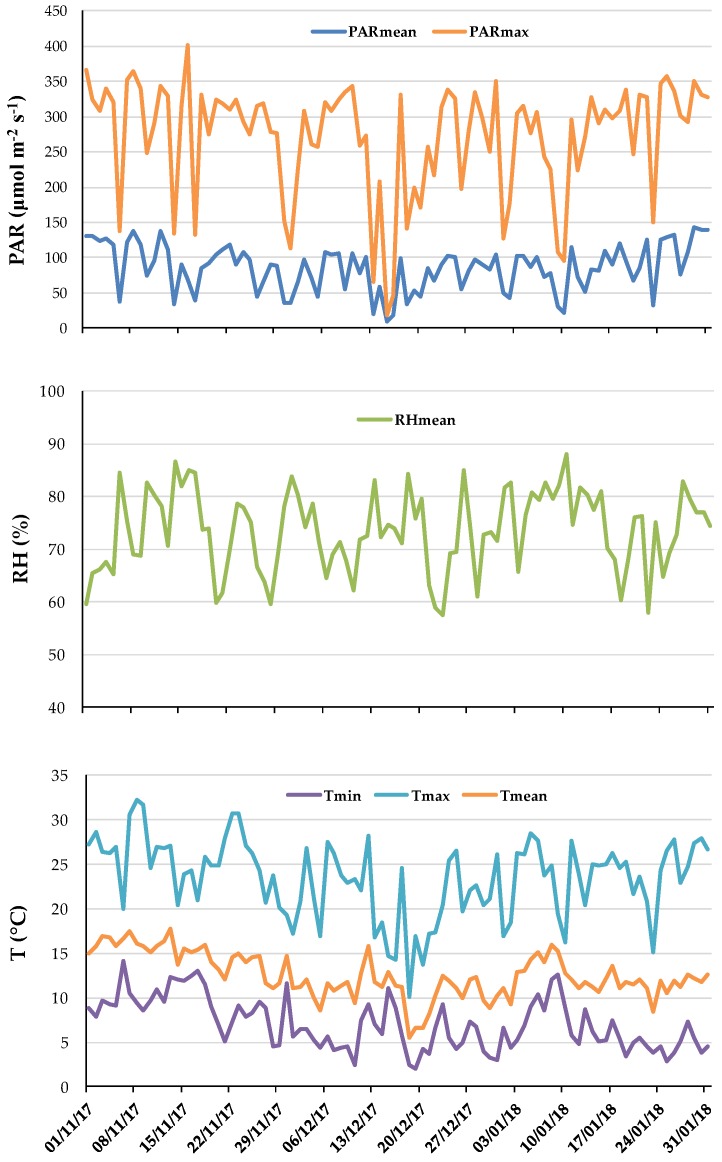
Photosynthetically Active Radiation (PAR), Relative Humidity (RH), average mean, minimum (min) and maximum (max) air temperatures (T), inside the greenhouse during the experiment.

Climatic parameters registered during the growing experiment are reported in Figure 1.

### 2.2. Yield and Sampling

After 21 day-iodine treatment (8 January 2018), the first harvest was carried out, collecting five plants per unit. After measurement of fresh weight and number of leaves, three sub-samples per unit were prepared from a bulk of leaves obtained for each unit: One sub-sample for the raw product characterization (leaf color, dry matter, nitrate, and I content) and the other two for cooking processing. After 22 days more (30 January 2018), the remaining five plants per unit were sampled in order to check the I content in the raw leaves, following the same bulk method used at the first harvest.

### 2.3. Cooking Procedure

Two sub-samples were allocated to the cooking trial, by boiling and steaming. After removing inedible parts, samples were washed with deionized water. The two cooking treatments (boiling and steaming) were applied for both the unbiofortified and biofortified vegetables. A total of 108 samples were prepared: 3 I treatments × 3 post-harvest treatments (raw, boiling, and steaming) × 4 genotypes × 3 replicates. Cooking conditions were determined by preliminary experiments carried out according to the judgement of a group of semi-trained panelists. For all cooking treatments, the minimum time to reach tenderness for an adequate palatability and taste, according to the Italian eating habits, was referred to Reference [21]. The cooking treatments were as follows.

Boiling: Samples were boiled in a steel pot with boiling distilled water (99.0 ± 1.0 °C) for 4 min at a vegetables/water volume ratio of 1:6. The samples were drained off and rapidly cooled on ice.

Steaming: Samples were placed on a tray in a steam cooker (VC 101 630 Tefal, Milan, Italy) covered with a lid and cooked with water vapor (99.0 ± 1.0 °C) for 8 min under atmospheric pressure. The samples were drained off and rapidly cooled on ice.

### 2.4. Measurements and Analyses

Color parameters were measured on raw sub-samples on 10 leaves per unit on the CIELAB scale 1976 (L*, a*, b*) with a portable tristimulus color-meter (Minolta Chroma Meter CR-400; Minolta Camera Co. Ltd., Osaka, Japan). The instrument runs with the color–space coordinates designed as: L*, the lightness value, ranging from black = 0 to white = 100; a*, ‘red/green chromaticity’, red–violet color if positive, green–blue color if negative; b*, ‘yellow/blue chromaticity’, yellow color if positive, blue color if negative. Through trigonometric functions, other color indices were calculated: (i) Color intensity or color saturation, C* = [(a*)^2^ + (b*)^2^]^1⁄2^; (ii) hue angle, h° = tan−1 (b*/a*) (where 0° = red-violet; 90° = yellow; 180° = blue-green; 270° = blue) [22].

A sub-sample of the raw material was oven-dried at 105 °C until constant weight for the determination of the dry matter content. Other sub-samples used for the raw product characterization were divided in two portions: One for freeze-drying and one for oven-drying (65 °C until constant weight). The first was used for I determination and the second for nitrate content in raw samples. Dry material was finely ground through a mill (IKA; Labortechnik, Staufen, Germany) with a 1.0 mm sieve and used for quantitative NO_3_^−^ analyses, following the method described in Reference [23] by ion chromatography (Dionex DX120, Dionex Corporation, Sunnyvale, CA, USA) with a conductivity detector, using a separation column IonPac AS14 and a pre-column IonPac AG14 (Dionex Corporation). The elution was performed using 3.5 mM Na_2_CO_3_ and 1 mM NaHCO_3_, and the flow rate was 1 mL/min at 35 °C, current 50 mA (Electrolytically regenerator Suppressor: Dionex AERS 500 4 mm RFIC). The quantification of nitrate in samples was determined by interpolation with a calibration curve, previously made with certified Element IC Standard (IC-MAN-18, CHEM-LAB).

For the inorganic I determination, we used the protocol reported in Reference [24]. Briefly, freeze-dried samples (raw and cooked) were taken and the I^−^ was extracted with hot water (60 °C) and stirred for 30 min. Then, the sample cooled down to room temperature. The product solution was well mixed and filtered through an ashless filter paper followed by a 0.2 μm membrane filter. After extraction, the colorimetric reaction was performed as reported by Reference [24]. Briefly, iodate standard solution and the extracts samples (100 µg/L) were treated with 1 mL of KSCN (0.023% *m*/*v*), 2 mL of NH_4_Fe(SO_4_)_2_ (7.7% *m*/*v*) in 2.4 M HNO_3_ and 2 mL of NaNO_2_ (0.02% *m*/*v*). The solutions were mixed and incubated in a water bath at 60 ± 2 °C for 1 h and subsequently incubated for 10 min in a water–ice mixture, in order to stop the colorimetric reaction. Each solution was read at 454 nm. The standards for inorganic I^−^ analysis were made from a 100 µg/L I^−^ stock solution, and standard concentrations ranged from 0 to 10 µg/L. The quantification of inorganic I^−^ in the freeze-dried samples was determined by interpolation with a calibration curve, previously made with an R^2^ = 0.9979. The accuracy and precision of I measurement procedures were verified by testing the certified reference standard 1573a—Tomato Leaves powder of the National Institute for Standards and Technology (NIST).

Iodine content in peat and perlite was lower than the quantification limit (LOQ = 0.15 mg/kg of dry sample) while the rain water used to prepare the NS contained 2.51 µg/L of I.

Iodine content was expressed on a Fresh Weight (FW) basis, in order to give an immediate quantification of the I intake from raw and cooked *Brassica* genotypes.

Nutrient solution-to-shoot transfer factor (TFshoot) was calculated as follows:

TFshoot = (ICshoot)_fresh_/IC_solution_

where (ICshoot)_fresh_ is the shoot I concentration on FW basis and IC_solution_ is the corresponding solution I concentration, according to that reported by Reference [25].

### 2.5. Statistical Analysis

Statistical analysis was carried out using the GLM (General Linear Model) procedure (SAS Software, Cary, NC, USA) by a split-plot experimental design in a two-way analysis of variance (ANOVA) for parameters measured on raw samples (yield and number of leaves, color, dry matter, and nitrate and I content) and by a split-split-plot design for I content determined on cooked leaves, compared to the raw ones. The least significant difference (LSD) test (α = 0.05) was used to establish differences between means.

## 3. Results

**Figure 2:**
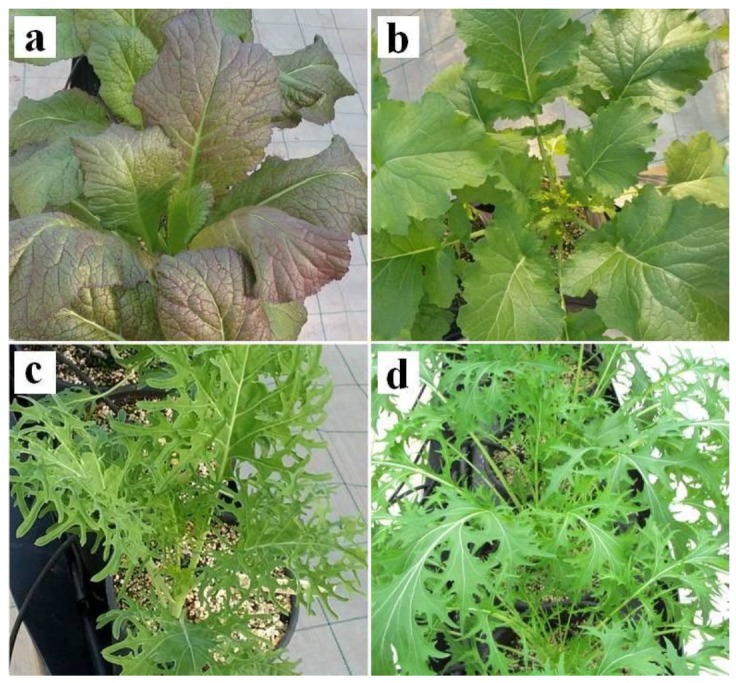
Plants of the four genotypes at the first harvest after 21 days of iodine biofortification. (**a**) red mustard; (**b**) broccoli raab; (**c**) curly kale; and (**d**) mizuna.

After 21 days of I biofortification, no toxicity effects were observed on *Brassica* plants regarding growth (Table 1) or morphological symptoms (Figure 2).

Shoot FW, as well as number of leaves, were modified only in relation to the different genotypes, since kale produced plants with the lowest FW (66% smaller than mizuna, which produced the biggest plants), while both kale and red mustard had the lowest number of leaves. On the other hand, broccoli raab and red mustard yielded shoot weights a bit lower than mizuna, but with a much lower number of leaves (mizuna produced a lot of thin leaves) (Table 1). Dry matter content was also not influenced by iodine biofortification. It was on average 7.1 and 9.8 g/100 g FW, respectively, at the first and second harvest (Table 2). 

**Figure 3:**
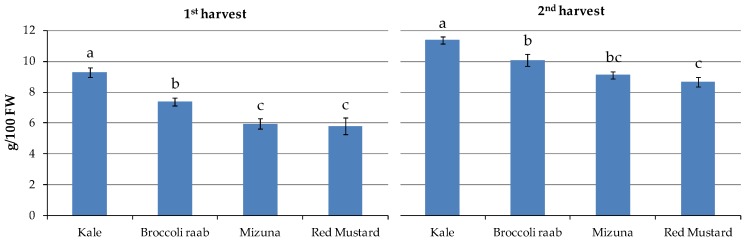
Effect of genotypes on DM measured at 1st and 2nd harvest (*p* ≤ 0.001). Different letters indicate that mean values are significantly different at α = 0.05 by LSD test.

The lowest mean values were found in red mustard (5.8 and 8.7 g/100 g FW) and mizuna (6.0 and 9.1 g/100 g FW), and the highest was in kale (9.3 and 11.4 g/100 g FW) at the two harvests, respectively, where red mustard and mizuna values were not statistically different from each other (Table 2; Figure 3).

**Figure 4:**
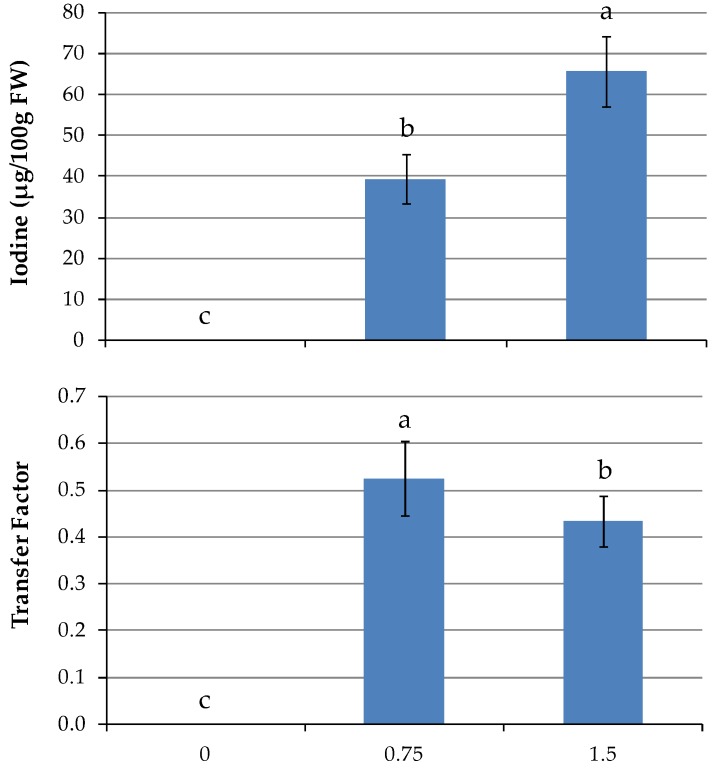
Effect of I rates in the nutrient solution on vegetable’s I content and TF at 2nd harvest (*p* ≤ 0.05). For I level 0 mg/L, values are lower than the quantification limit. Different letters indicate significant differences at α = 0.05 by LSD test.

After 21 days of biofortification, I accumulation in the edible raw product reached average values of 18.9 and 35.7 µg/100 g FW, respectively, with 0.75 and 1.5 mg/L (Table 2). Only in kale was there no increase in I accumulation by doubling rate from 0.75 to 1.5 mg/L. In the other species, I increased by 52, 134, and 137%, respectively, in mizuna, broccoli raab, and red mustard (Table 2). The highest I content was found in broccoli raab at the highest level of I in the NS. At the second harvest, I content was not different between the genotypes; on the other hand, I rates had effects more pronounced, with 39.3 and 65.7 µg/100 g FW, respectively, corresponding to the lower and higher I concentrations in the NS (Table 2; Figure 4). As expected, taking into account the low levels of accumulated I, the calculated transfer factor from the NS to shoot (TF_shoot_) was quite low in *Brassica*, on average 0.26 and 0.53 with 0.75 mg/L (5.9 µM), at the first and second harvest, respectively. The values were slightly lower at 1.5 mg/L (11.8 µM) (Table 2; Figure 4).

Color parameters were influenced only by genotypes, indeed deeply different for leaf color. Main differences regarded lower L* for kale (darker leaves), compared to the other three genotypes (33.5 versus 37.1), and a* (−18.0%), *h°* (−6.6%) and C* (−15.8%) values in red mustard lower than in green *Brassica* genotypes due to the reddish color of leaflet. No color parameter was modified by I biofortification (Table 3).

**Figure 5:**
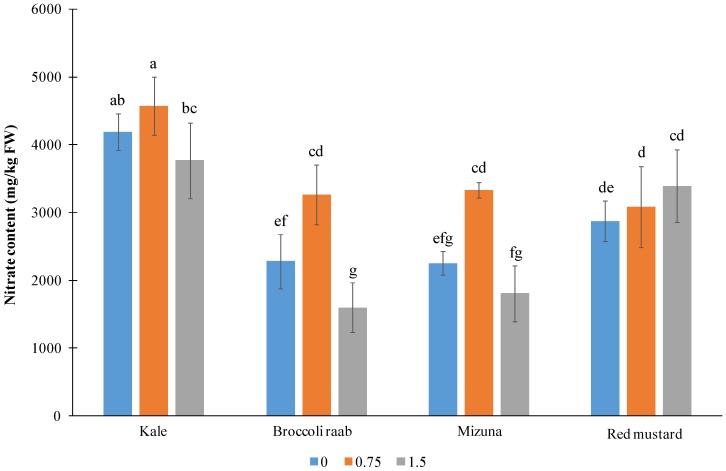
Nitrate content in samples of four *Brassica* genotypes grown with three I levels (mg/L) in the NS. Interaction significance: *p* ≤ 0.01. Different letters indicate significant differences at α = 0.05 by LSD test.

The highest NO_3_^−^ content was found in kale leaves (mean > 4000 mg/kg FW) (Figure 5).

The average NO_3_^−^ content measured in the other genotypes was in the range of 2400 to 3100 mg/kg FW. Interestingly, the lowest NO_3_^−^ content in kale, broccoli raab, and mizuna was measured with an I application of 1.5 mg/L (respectively, lower by 18, 51, and 46% than the content found at the intermediate I rate). The nitrate content was independent on I treatment only in red mustard, varying casually around 3000 mg/kg FW (Figure 5). In broccoli raab and mizuna, the highest accumulation was observed in plants treated with 0.75 mg/L of I (3260 and 3340 mg/kg FW, respectively), while in kale, the corresponding value was not statistically different from the untreated control.

**Figure 6:**
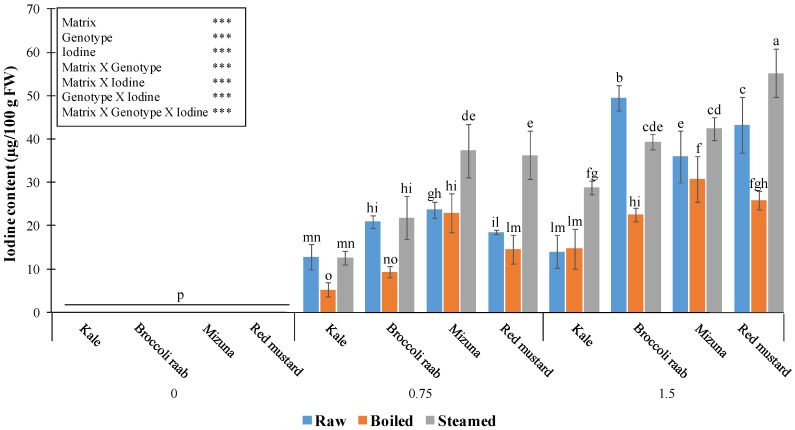
Iodine content in raw and cooked samples of four *Brassica* genotypes grown with three I levels (mg/L) in the nutrient solution. For I level 0 mg/L, values are lower than the quantification limit. Different letters indicate significant differences at α = 0.05 by LSD test. In the box in the upper side of the figure, the statistical results (***: significant at *p* ≤ 0.001) corresponding to the main factors and interactions are reported. Matrix stands for type of material (raw, boiled, and steamed).

Analytical and statistical results about the effect of the two cooking methods, genotypes, and I levels on shoot I content are given in Figure 6.

Steaming always increased the final I amount in mizuna and red mustard, compared to the raw products, and up to doubled values in red mustard supplied with 0.75 mg/L. In kale and broccoli raab, steaming did not change I content at the application of 0.75 mg/L but doubled it in kale at 1.5 mg/L (Figure 6). Only in broccoli raab supplied with 1.5 mg/L was there a 20.5% decrease of I content in the steamed, compared to the raw, product (Figure 6). Four min boiling always reduced I content, compared to the raw vegetables (in some cases more than halved), except for kale biofortified with 1.5 mg/L I, and mizuna and red mustard treated with 0.75 mg/L I (Figure 6).

## 4. Discussion

In our experiment, I treatment had no toxicity effects on the *Brassica* plants after either 21 or 43 days. This was ascertained through morphological, colorimetric, and biomass production observations. Indeed, we did not expect negative effects of I biofortification on biomass production, since we chose to apply very low doses of I (5.9 and 11.8 µM). In comparison, other studies applied quite high levels of I, up to 39, 80, or 100 µM to Chinese cabbage, lettuce, or spinach, respectively [25,26,27]. In Chinese cabbage, growth was depressed as early as at 0.5 mg/L (3.9 µM), and iodide was more effective than iodate in the depressing effect [26]. In lettuce iodate increased the biomass production, different than iodide [27]. Spinach biomass was not influenced by iodate, even at high doses up to 100 µM, while iodide was generally depressed growth [25]. The iodide form is actually more effective than iodate in biofortification processes but has a detrimental influence on plant growth, mainly due to its excessive accumulation in plant tissues, since it is directly taken up by plant roots [11]. On the contrary, iodate has to be reduced to iodide before its uptake [11]. These findings were at the base of our choice to use iodate as the I form for *Brassica* biofortification. Regarding color alterations, similar to other studies about biofortification of vegetables [28,29], our results evidenced no changes of color, notoriously considered the first quality parameter evaluated by consumers and, at the same time, the indicator of a chlorophyll status change. Similarly, chlorophyll and carotenoid contents were not changed by I fertilization (under any form) in lettuce [12], probably due to the low I concentration in treatments, while I excessive fertilization can lead to leaf chlorosis, in the older leaves more than in the young, driven by transpiration [30].

Compared to other studies, where higher I doses were applied (and recalculating I values, correspondingly), the I concentrations in *Brassica* leaves appeared low: 7.8 mg/kg Dry Weight (DW) was the highest value found in broccoli raab. This was a good value for our aims, but decisively low if compared to the awfully high values up to 50 mg/kg DW accumulated in lettuce when an iodate level similar to our treatments (1 mg/L) was applied in Nutrient Film Technique (NFT) [12]. If supplied with 20 µM (2.54 mg/L) of I from KIO_3_, lettuce leaves accumulated 566 mg/kg DW [26]. Probably lettuce is a sensitive species, since its leaves also accumulated 8.1 mg/kg DW of I as maximum value [11] when exposed to very low rates of I in NS (up to 129 µg/L from KIO_3_). Based on our results, in *Brassica* genotypes, I uptake and translocation to leaves may be comparably lower. Our *Brassica* at 1.5 mg/L of I-IO_3_ accumulated 0.66 mg/kg FW (on average of four species) (Table 2). However, this value was only the 30^th^ part of what (about 20 mg/kg FW) was found in Chinese cabbage with the application of 1 mg/L of I-IO_3_ in solution culture [26]. The duration of treatments on Chinese cabbage is unclear, since the authors reported a growing cycle duration of three months when referring to a different pot experiment. Correspondingly, they observed toxicity effects and growth reduction at an iodate concentration of 0.5 mg/L. These effects did not appear under our conditions—not after 21 days of treatments nor after an additional 22 days, when we did an additional sampling to check I accumulation and toxicity occurrence (Table 2). Specifically, at 43 days of biofortification, I accumulation in the edible product further increased compared to the previous harvest, with nearly doubled values. On the one hand, we could state that I accumulation was dependent on genotype aptitude, but, at the same time, it depended on the complex of the features that characterized the biofortification (applied I doses and form, supply protocol, and cultivation conditions). One aspect may be specifically crucial for I accumulation: The interference, or not, of a substrate influencing the I fate at root level [16]. Our cultivation could be ascribed to a soilless culture, where the direct mean to supply I is fertigation with an NS; however, in our experiment, plants were grown in pots containing peat in a mixture with perlite (1:3 volume ratio peat:perlite). It is known that organic matrices absorb I from the circulating solution [31]. This aspect may act in a double direction: increasing availability of I over time, covering a function of accumulation, or subtracting I from the root zone during fertigation [16]. It is sure that our plants were not in continuous contact with the NS, different than NFT or solution culture. Under these conditions, comparing our values to the TF_leaf_ (about 11.6) reported by Reference [25] for spinach supplied with a similar I concentration for 21 days, it is clear that the uptake and translocation efficiency of I-IO_3_ in the NS to the *Brassica* shoot appeared very low. Expressing these values in percentage, we can state that maximum 50% of the supplied I was taken up by *Brassica* plants. Nevertheless, it was noteworthy that, from the first to the second harvest, the TF of our plants was almost doubled in some cases. This could be due to an increased root volume, a higher I amount accumulated in the substrate, or simply a longer exposure to I supply.

The nitrate concentrations found in this study are reasonable values, considering that *Brassica* are medium nitrate accumulating species [32]. In literature, there are opposite findings about the effect of I on nitrogen uptake and nitrate accumulation in vegetables, especially regarding the I form. Iodate could limit or depress nitrification (conversion of ammonium to nitrate) and denitrification (conversion of nitrate to N_2_) in the soil. Smolen and colleagues [31] attributed the higher availability of nitrogen for carrot root uptake to this effect of iodate, compared to iodide supply, but nothing is known about carrot nitrate accumulation. Conversely, Signore et al. [33], applying I by leaf spraying, found that iodate biofortification had a different effect on nitrate accumulation in carrot roots depending on I rates: both low (50 mg/L) and high (500 mg/L) levels decreased nitrate content, but the first had a stronger effect. Finally, supplied by NS, iodate did not change the nitrate content in lettuce, different than biofortification with iodide. Probably, iodate induced a high absorption of nitrate but, at the same time, stimulated nitrate reductase and nitrogen assimilation (consequently nitrate did not accumulate in leaves); on the contrary, iodide decreased both the concentration of nitrate and the nitrate reductase activity [34]. Definitely, few studies have been carried out regarding the effect of I on nitrogen nutrition and nitrate accumulation to give a certain answer to this question. An additional piece of information is that iodate can be reduced to iodide by nitrate reductase, but this has been observed in extracts from phytoplankton, and not from corn seedlings [35]. It is not clear if nitrate reductase from other terrestrial plants can be effective and how iodate and nitrate interfere in this process [16]. A general hypothesis could be stated that iodate somehow influences nitrogen metabolism and nitrate accumulation, depending on the I dose, plant species, and interactions with other anions in the root environment [15].

Considering that the four *Brassica* genotypes are eaten as cooked vegetables, in order to assess I intake after cooking, we determined the I content in the edible product cooked by boiling and steaming. The effect of boiling was expected, as supported by some evidences in literature. For example, biofortified celery leafstalks lost 0.7% and 42.5% iodine after 2 and 30 min boiling at 100 °C, respectively [13]. Other results reporting I losses after boiling regard not leafy vegetables, such as carrot, potato, or tomato [17,20]. Steaming losses of I are generally more contained than boiling [19], similarly to other bioactive compounds measured in *Brassica* vegetables [36].

Given an I concentration of 36 µg/100 g FW, found as average of the four studied *Brassica* genotypes, and under the hypothesis that we would eat them raw, we will cover 24% of the I RDA referred to an adult (150 µg/day) through the consumption of only 100 g of fresh product. This percentage would be decreased or increased in the case of boiled or steamed product consumption, respectively. When calculated on 43 day-biofortified vegetable products, 100 g of *Brassica* would provide, on average, 66 µg I, corresponding to 44% of RDA. Iodine biofortification of *Brassica* may be helpful in increasing I intake in Asian diets, which are rich in these vegetable species, and at the same time, not comprehensive of iodate fortified salt that is not used as dietary condiment. Definitely, the accumulation rate and the biofortification efficiency were very low in the studied *Brassica* under our conditions, compared to other studies, but this result gives an optimal margin of safety in reference to the risk of excessive dietary intake (taking into account all sources, from drinking water to iodized salt to vegetables, fish, and seafood) of I that can cause similar damage as I deficiency [37].

## 5. Conclusions

A higher I rate generally increased I content in all *Brassica* genotypes, without toxicity effects from the I, in terms of reduced growth or morphological symptoms, after both 21 and 43 day-I biofortification.

The maximum I content was reached in broccoli raab shoots (49.5 and 75.7 µg/100 g FW, respectively, in the two harvests), much lower than values found in literature for leaf vegetables. Our choice to apply low levels of I for biofortifying *Brassica* leafy vegetables proved to be rational since it did not damage plants and avoided the risk to overcome the recommended dose for human adult intake of 150 µg/day and to occur in cases of human toxicity. At the accumulated I contents at the second harvest, a bit more than 200 g of raw *Brassica* leaves would be enough to cover the recommended dose.

The higher I rate (1.5 mg/L) did not change the nitrate content, except for broccoli raab where it was reduced. On the other hand, the lower I dose never reduced, or rather raised (broccoli raab and mizuna), the nitrate accumulation, compared to untreated plants, the effect being dependent on genotypes.

Cooking can change I content depending on genotype but also on cooking methods. Generally, boiling reduced I content, while steaming increased it or left it unchanged. Therefore, the cooking method should be chosen in order to preserve and/or enhance the I final amount in the cooked product.

Deeper study should be made about the interference of I with nitrate, especially on nitrate reductase activity.

## Figures and Tables

**Table 1:** Biometric parameters of *Brassica* genotypes supplied with different levels of I in the nutrient solution.

Genotype	Shoot Fresh Weight	No. of Leaves
	g/plant	n./plant
Kale	128.8 d	12.1 c
Broccoli raab	327.4 b	19.0 b
Mizuna	378.4 a	81.8 a
Red Mustard	299.5 c	11.0 c
**Iodine (mg/L)**		
0	287.0	30.5
0.75	293.5	32.0
1.5	270.2	30.4
**Significance**		
Genotype	***	***
Iodine	ns	ns
Genotype × Iodine	ns	ns

*** Significant at *p* ≤ 0.001; ns: not significant. Different letters within columns indicate significant differences at α = 0.05 by least significant difference (LSD) test.

**Table 2:** Dry matter (DM), I concentration, and transfer factor (TF) of *Brassica* genotypes supplied with different levels of I in the nutrient solution (NS), measured after 21 and 43 days after iodine biofortification (I and II harvests).

Genotype	NS Iodine		I Harvest (08/01/2018)			II Harvest (30/01/2018)	
		DM	Iodine	TF	DM	Iodine	TF
	mg/L	g/100 g FW	µg/100 g FW		g/100 g FW	µg/100 g FW	
Kale	0	9.40	ND g	--	11.27	ND	--
	0.75	9.52	12.73 f	0.17	11.67	39.23	0.52
	1.5	8.97	13.98 ef	0.09	11.23	68.13	0.45
Broccoli raab	0	7.16	ND g	--	10.40	ND	--
	0.75	7.32	20.87 d	0.28	10.20	34.97	0.47
	1.5	7.65	49.47 a	0.33	9.67	75.70	0.50
Mizuna	0	5.84	ND g	--	9.13	ND	--
	0.75	5.71	23.64 d	0.32	8.90	35.23	0.47
	1.5	6.33	35.89 c	0.24	9.37	55.10	0.37
Red mustard	0	5.47	ND g	--	8.63	ND	--
	0.75	5.51	18.46 de	0.25	8.37	47.90	0.64
	1.5	6.43	43.24 b	0.29	9.03	63.70	0.42
**Significance**							
Genotype		***	***	***	***	ns	ns
Iodine		ns	*	**	ns	*	*
Genotype × Iodine		ns	***	***	ns	ns	ns

* Significant at *p* ≤ 0.05; ** Significant at *p* ≤ 0.01; *** Significant at *p* ≤ 0.001; ns: not significant. Different letters within columns indicate significant differences at α = 0.05 by LSD test. NS: Nutrient Solution; FW: Fresh Weight; ND: Not Detected (value < quantification limit).

**Table 3:** Color parameters of raw leaves in *Brassica* genotypes supplied with different levels of I in the NS.

Genotype	L*	a*	b*	h°	C
Kale	33.53 b	−14.04 b	17.56	128.69 a	15.24 a
Broccoli raab	37.62 a	−14.36 b	17.85	128.87 a	15.55 a
Mizuna	36.85 a	−13.18 b	16.33	128.91 a	14.36 ab
Red Mustard	36.78 a	−11.36 a	19.22	120.33 b	12.96 b
**Iodine**					
0	35.83	−12.94	17.49	126.28	14.26
0.75	36.65	−13.40	17.91	126.88	14.68
1.5	36.10	−13.37	17.83	126.94	14.65
**Significance**					
Genotype	***	**	ns	***	**
Iodine	ns	ns	ns	ns	ns
Genotype × Iodine	ns	ns	ns	ns	ns

** Significant at *p* ≤ 0.01; *** Significant at *p* ≤ 0.001; ns: not significant. Different letters within columns indicate significant differences at α = 0.05 by LSD test.
